# Antioxidant Activity of Polysaccharide-enriched Fractions Extracted from Pulp Tissue of *Litchi Chinensis* Sonn.

**DOI:** 10.3390/molecules15042152

**Published:** 2010-03-25

**Authors:** Fanli Kong, Mingwei Zhang, Sentai Liao, Shujuan Yu, Jianwei Chi, Zhencheng Wei

**Affiliations:** 1 Key Laboratory of Functional Food, Ministry of Agriculture, Bio-tech Research Institute, Guangdong Academy of Agricultural Sciences, Guangzhou 510610, China; 2 College of Light Industry and Food Science, South China University of Technology, Guangzhou 510640, China

**Keywords:** antioxidant activity, litchi, polysaccharide, functional food

## Abstract

Natural antioxidants such as polysaccharides with strong antioxidant activities are used to protect against oxidative damage, yet little is known so far about the antioxidant effects of litchi fruit polysaccharides. In the present study, four different polysaccharide-enriched fractions were isolated from litchi pulp tissue and partially purified by a stepwise method of ethyl alcohol (EtOH) precipitation. Their chemical and physical characteristics were determined by chemical methods, gas chromatography and IR spectrophotometry. Antioxidant activities of these fractions were investigated using various *in vitro* assay systems. These four polysaccharide-enriched fractions exhibited a dose-dependent free radical scavenging activity as shown by their DPPH radical, superoxide anion and hydroxyl radical inhibition, chelating ability and reducing power. Among the different fractions, LFP-III showed the strongest scavenging activity against DPPH radical, superoxide and hydroxyl radicals and chelating ability. These findings suggest litchi polysaccharides from pulp tissue have potential as functional foods with enhanced antioxidant activity.

## 1. Introduction 

Oxidation is an essential process for all living organisms for the production of energy necessary for biological processes [1]. In addition, oxygen-centered free radicals are involved in development of a variety of diseases, including cellular aging, mutagenesis, carcinogenesis, coronary heart disease, diabetes, and neurodegeneration [2,3]. Though almost all organisms possess antioxidant defense and repair systems to protect against oxidative damage, these systems are often insufficient to prevent the damage entirely [4]. Recently, much attention was paid to screening natural biomaterials in the case of several clinical situations since use of synthetic antioxidants is restricted due to their carcinogenicity [5]. Among various natural antioxidants, polysaccharides in general have strong antioxidant activities and can be explored as novel potential antioxidants [6,7]. As reported recently, polysaccharides isolated from fungal, bacterial and plant sources were found to exhibit antioxidant activity, and were proposed as useful therapeutic agents [8,9,10,11].

Litchi (*Litchi chinensis* Sonn.), a tropical to subtropical fruit originally from China is cultivated all over the world in warm climates [12]. Litchi fruits have been used to produce various types of healthy products and foods, e.g., medicinal beverages, drinks or soup [13,14,15]. This suggests that it merits investigation as a potential antioxidant for clinical use. Polysaccharide from litchi pericarp tissues has been purified and the antioxidant activities evaluated [16], and this demonstrated its beneficial antioxidant properties. However, it is not known whether its pulp tissue has any direct antioxidant effects and more detailed work is required to fully elucidate the antioxidant activities of the active polysaccharides.

Some methods were reported previously for polysaccharide concentration and purification,. For example, ultrafiltration was used to concentrate and classify polysaccharides into different molecular weight ranges by varying embrane diameter [17]. Another way was using different concentrations of salt solutions (e.g. ammonium sulfate) to precipitate polysaccharides [18]. The most established and easiest was the stepwise ethyl alcohol (EtOH) precipitation method using different concentrations: first a low concentration of EtOH for large MW polysaccarides, then a high concentration for small MW polysaccharides [19]. 

In the present study, different polysaccharide-enriched fractions were isolated from pulp of litchi and partially purified by the stepwise EtOH precipitation method. Their chemical and physical characteristics were determined by chemical methods, gas chromatography and IR spectrophotometry Their possible antioxidant activities were then investigated using various *in vitro* assay systems such as DPPH radical scavenging, superoxide radical scavenging, hydroxyl radical scavenging, and ferrous ion chelating ability and reducing power, in order to understand the potential usefulness of litchi pulp as a functional food. 

## 2. Results and Discussion

### 2.1. Chemical composition of polysaccharides

**Figure 1:**
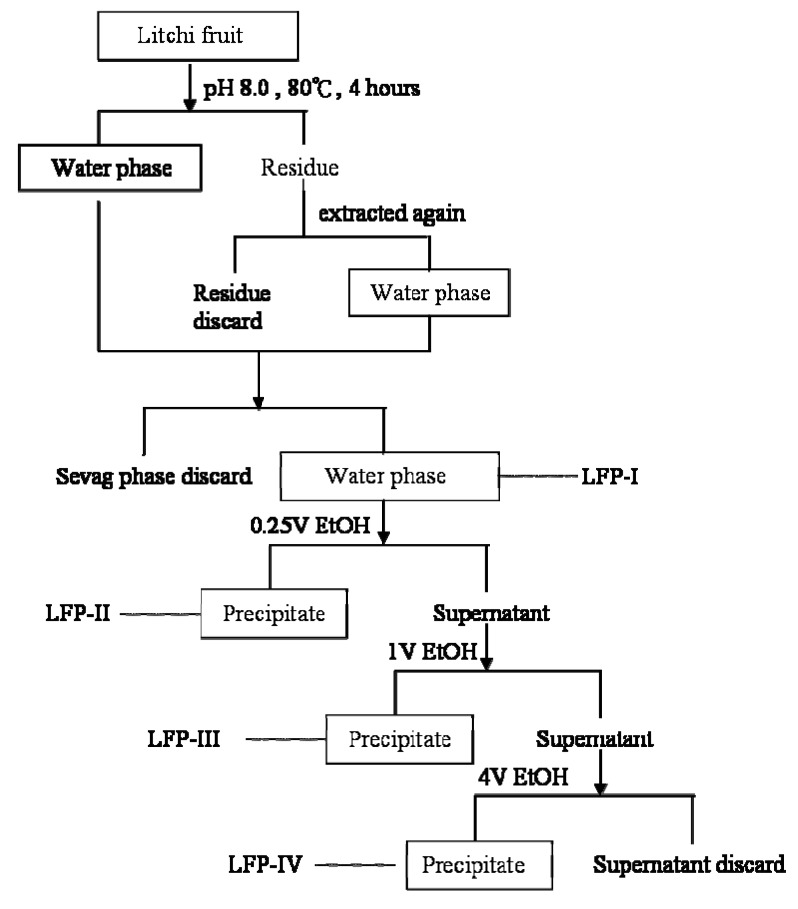
Flow sheet for extraction and fractionation polysaccharides from litchi pulp tissue by EtOH stepwise precipitation.

Byfollowing the procedures summarized in the flow chart of Figure 1, four polysaccharide-enriched fractions were extracted from litchi pulp. The carbohydrate content, protein content, and sugar compositions of LFP-I, LFP-II, LFP-III and LFP-IV were determined and are given in Table 1. The carbohydrate contents increased from LFP-I to LFP-IV, and the respective yields were 79.21%, 85.15%, 93.09% and 96.77% (wt%). 

The protein contents of the different polysaccharide fractions were LFP-I 2.81%, LFP-II 1.31%, LFP-III 4.24%, LFP-IV 1.23%, respectively. GC analysis of the trimethylsilyl derivatives showed differences between these four fractions, with the presence of arabinose, rhamnose, ribose, galactose and glucose in the molar ratio of 1.95:2.00:1.00:2.04:1.57 for LFP-I, arabinose, ribose and glucose in the molar ratio of 1.00:1.20:1.47 for LFP-II, arabinose, rhamnose, ribose, galactose and glucose in the molar ratio of 1.30:1.91:1.54:2.13:1.00 for LFP-III, and arabinose, rhamnose, galactose and glucose in the molar ratio of 1.60:1.00:1.07:1.21 for LFP-IV.

### 2.2. IR spectra of the four LFP fractions

The infrared spectra of the four LFP fractions displayed a characteristic broad intense stretching peak at around 3,263 cm^-1^ for the hydroxyl group, and a C-H stretching band at around 2,937 cm^-1^ [20]. The two peaks towards 1,727 and 1,420 cm^-1^ in the IR spectra resulted from the presence of the COO-deprotonated carboxylic group [21]. A band of absorption at around 771cm^-1^ represents the symmetrical ring vibrations for the four LFP fractions [22].

### 2.3. DPPH radical-scavenging activity

**Figure 2:**
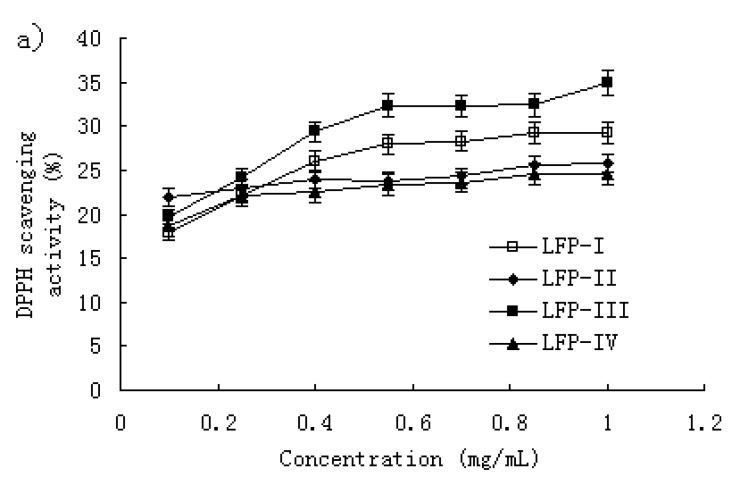

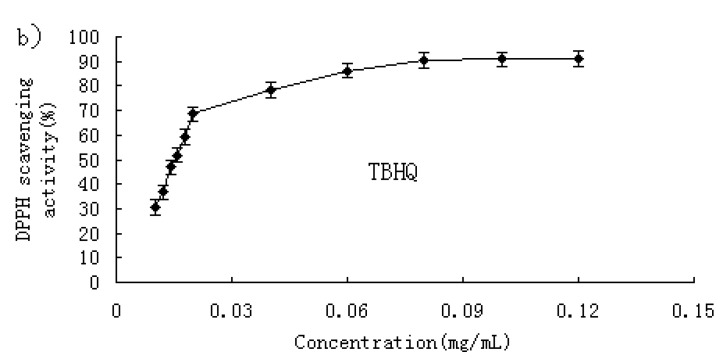
(a) Scavenging effect of LFPs on DPPH radicals. Each value is presented as mean ± standard error (n=3). (b) Scavenging effect of TBHQ on DPPH radicals. Experimental results were means ± standard error of three parallel measurements.

The DDPH radical-scavenging activity has been extensively used to screen antioxidants from fruit and vegetable juices or extracts [23,24,25]. Figure 2a shows the DPPH radical scavenging activity of the litchi fruit polysaccharide fractions. For LFP-I and LFP-III, at the concentration below 0.55 mg/mL, the scavenging effect increased with increasing concentration, but slowly; at the concentration of 1.0 mg/mL, the scavenging effect was about 29.2% and 34.9% respectively. For LFP-II and LFP-IV, the scavenging effect showed no significant increase with increasing concentration; at 1.0 mg/mL, the scavenging effects were about 25% and 24%, respectively. 

In addition, the scavenging effect of TBHQ against DPPH radical was studied using the same model. As shown in Figure 2b, the TBHQ significantly inhibited activity of DPPH radicals in a dose-dependent manner; at 1.0 mg/mL, the scavenging effect was about 90.8%. As comparison, TBHQ showed stronger scavenging activity for DPPH radical than the LFPs (P < 0.01).

### 2.4. Superoxide anion-scavenging activity

Superoxide anion plays an important role in plant tissues and is involved in the formation of other cell-damaging free radicals [26]. The superoxide radical is known to be produced *in vivo* and can result in the formation of H_2_O_2_ *via* a dismutation reaction. Moreover, the conversion of superoxide and H_2_O_2_ into more reactive species, e.g., the hydroxyl radical, has been thought to be one of the unfavorable effects caused by superoxide radicals. The superoxide radical scavenging effect of LFPs and Vitamin C (Vc) was tested in a PMS/NADH system for being assayed in the reduction of NBT [27]. 

**Figure 3:**
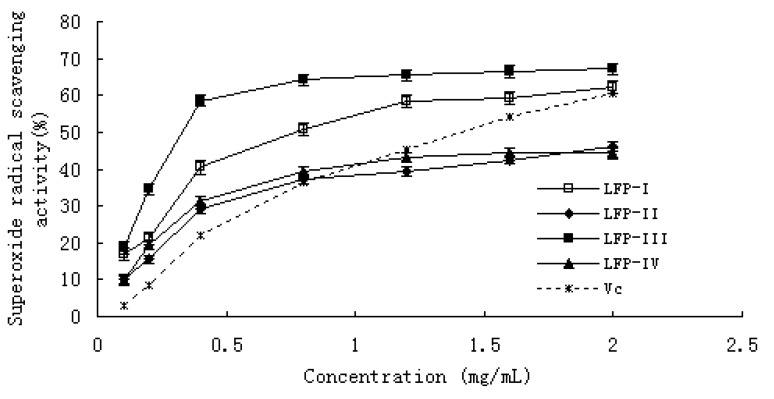
Scavenging effect of LFPs on superoxide radicals. Each value is presented as mean ± standard error of three parallel measurements for each concentration.

The LFPs’ and Vc inhibition of the activity of superoxide radical in a dose-dependent manner is shown in Figure 3. At a concentration from 0.1 to 2.0 mg/mL, the scavenging effect was 17.10–62.07% for LFP-I, 10.28–46.24% for LFP-II, 18.62–67.24% for LFP-III, 9.62–44.24% for LFP-IV, and 3.12–60.34% for Vc, respectively. The IC_50_ values of LFP-I, LFP-III and Vc were 1.29, 0.87 and 0.49 mg/mL; however, the IC_50_ values of LFP-II and LFP-IV could not be read in Figure 2. Compared with this result, LFP-I and LFP-III showed a stronger scavenging activity for superoxide radicals than Vitamin C (p < 0.05). The antioxidant activity of LFP-II and LFP-IV is stronger than that of Vc at the dosage range of 0–0.8 mg/mL, but with continually increasing concentration, the scavenging effect of the polysaccharides becomes weaker than that of Vc. These results clearly showed that the antioxidant activities of all samples were related to the superoxide radical scavenging abilities.

### 2.5. Hydroxyl radical scavenging activity

**Figure 4:**
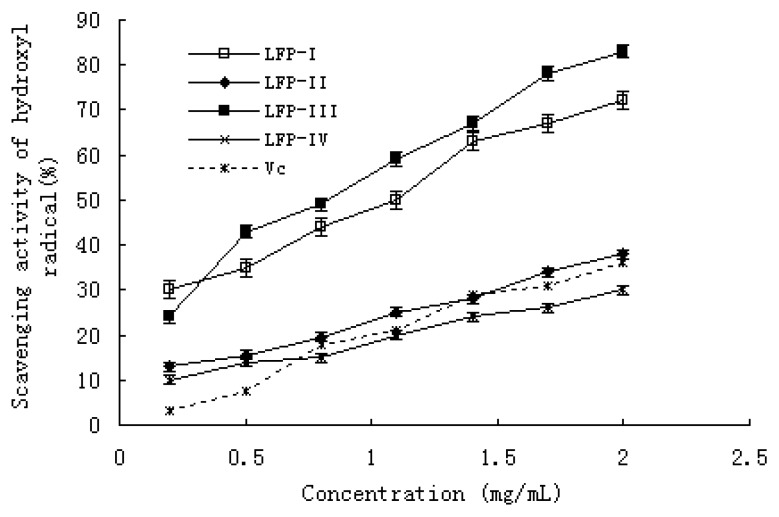
Scavenging effect of LFPs on hydroxyl radicals, using the control of Vc. Data represent the mean ± standard deviation (n = 3).

The hydroxyl radical generated in the system by the Fenton reaction, is known to be the most reactive of all the reduced forms of dioxygen and is thought to initiate cell damage *in vivo* [28]. The scavenging effects of LFPs and Vitamin C are shown in Figure 4. Among the four samples and Vitamin C, LFP-I and LFP-III had stronger scavenging effect against hydroxyl radical. At the same time, the scavenging effect of all LFPs and Vitamin C increased with increasing the concentration. At a concentration of 0.2–2.0 mg/mL, the scavenging effect was 30.11–72.24% for LFP-I, 13.22–38.15% for LFP-II, 24.15–83.46% for LFP-III, 10.26–30.18% for LFP-IV and 3.23–36.31 for Vitamin C. The IC_50_ values of LFP-I and LFP-III were 0.81 and 0.65 mg/mL, and the IC_50_ values of LFP-II, LFP-IV and Vc could not be read. 

**Figure 5:**
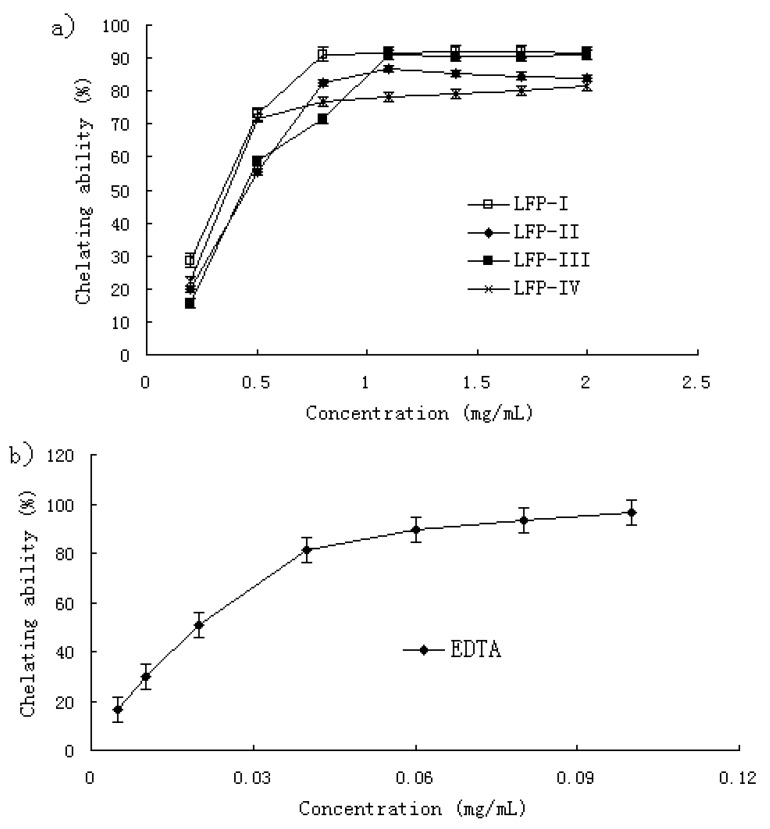
(a) Chelating ability of LFP. Each sample was assayed in triplicate for each concentration. Each value is presented as mean ± standard error. (b) Chelating ability of EDTA. Each value is presented as mean± standard error (n = 3).

For hydroxyl radical, there are two types of antioxidation mechanism: one suppresses the generation of hydroxyl radical, and the other scavenges the hydroxyl radicals generated [29]. In the former, the metal ions may react with H_2_O_2_ to give hydroxyl radicals and form the corresponding metal complexes, while the antioxidant activity may bond to these metal ions and thus cannot further react with H_2_O_2_. In another assay system in this study, we demonstrated the iron chelating ability of all samples. As shown in Figure 5, all the LFPs exhibited strong chelating ability. It was likely that the radical scavenging activity of LFPs can be attributed to not only the chelating activity, but also their scavenging of the hydroxyl radicals generated.

### 2.6. Ferrous ion chelating ability 

Fe^2+^ ion is the most powerful pro-oxidant among various species of metal ions [30]. Ferrozine can quantitatively form complexes with Fe^2+^. In the presence of other chelating agents, the complex formation is disrupted with the result that the red color of the complexes decreases. Measurement of color reduction therefore allows the estimation of the metal chelating ability [31,32]. It was reported that chelating agents, which form σ bonds with a metal, are effective as secondary antioxidants because they reduce the redox potential, thereby stabilizing the oxidized form of the metal ion [33]. 

The chelating ability of LFPs and EDTA was concentration-related as shown in Figure 5a and Figure 5b. All LFPs showed strong chelating ability. At a concentration of 0.2–2.0 mg/mL, the chelating ability was 28.57–91.53% for LFP-I, 20.0–83.75% for LFP-II, 15.84–90.50% for LFP-III and 22.45–81.33% for LFP-IV. However, as shown in Figure 5b, the chelating ability of all samples was weaker when compared with that of EDTA. Metal chelating activity was significant as they reduced the concentration of the catalyzing transition metal in lipid peroxidation [34]. Our data on the chelating ability of LFPs reveal that the polysaccharides demonstrate an effective capacity for iron binding suggesting that their action as antioxidant may also be related to their iron-binding capacity.

### 2.7. Reducing power

**Figure 6:**
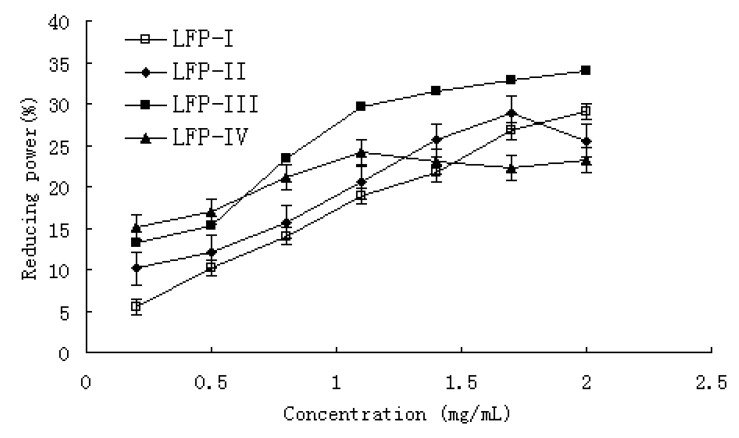
Reducing power of LFPs. Each sample was assayed in triplicate for each concentration. Experimental results were means ± SD of three parallel measurements. Each value is presented as mean± standard error (n = 3).

It has been reported that reducing power is associated with antioxidant activity and may serve as a significant reflection of the antioxidant activity. The presence of reductants such as antioxidant substances in the antioxidant samples causes the reduction of the Fe^3+^/ferricyanide complex to the ferrous form. Therefore, Fe^2+^ can be monitored by measuring the formation of Perl’s Prussian blue at 700 nm [35]. As shown in Figure 6, the reducing power of all LFPs correlated well with increasing concentration. At a concentration of 0.2–2.0 mg/mL, the reducing power was 5.50–29.10% for LFP-I, 10.15–25.56% for LFP-II, 16.22–43.89% for LFP-III and 15.12–23.17% for LFP-IV, respectively. When the concentration is higher than 0.65 mg/mL, the reducing power of LFP-III was the highest among the four different LFPs. There is a direct correlation between antioxidant activities and reducing capacity of certain plant extracts [36]. The reducing properties are generally associated with the presence of reductones, which have been shown to exert antioxidant action by breaking the free radical chain by donating a hydrogen atom [37]. Reductones are also reported to react with certain precursors of peroxide, thus preventing peroxide formation. Though the result of this study was weaker than Vc, it showed that reducing power of four different fractions also probably plays a role in the observed antioxidation.

## 3. Experimental

### 3.1. Materials 

Fresh fruits of Litchi (*Litchi chinesis* Sonn.) cv. Heiye at the commercially mature stage were picked from a commercial orchard in Guangzhou, China. Fruits of uniform shape and color were selected as materials. Pulp tissue of litchi was collected and used as extracted materials.

### 3.2. Chemicals

All chemicals used were of analytical grade. 1,1-Diphenyl-2-picryldydrazyl (DPPH), nitro blue tetrazolium (NBT), phenazine methosulfate (PMS), dihydronicotineamidadenine dinucleotide (NADH), tert-butylhydroquinone (TBHQ) were purchased from Sigma Chemical Co. (St Louis, MO, USA). 

### 3.3. Preparation of litchi pulp polysaccharide-enriched fractions (LFPs)

LFPs were prepared by stepwise methods according to the methods of Miyazaki and Nishijima [38] and Olafsdottir [39] with some modifications. A summary flow chart was shown in Figure 1. Litchi pulp tissue (1 kg) was refluxed three times with 80% EtOH to remove monosaccharides and saccharoses. Then it was comminuted and extracted twice with 1 Liter portions of distilled water for 4 h at 80 °C. The water extracts were filtered through Whatman No. 1 paper. The filtrates were combined and concentrated to 250 mL by a rotary evaporator at 40 °C. The proteins in the extract were removed using the Sevag reagent (see Figure 1). The supernatants were evaporated to remove Sevag reagent, then concentrated and lyophilized to afford LFP-I. The water extracts were precipitated with 0.25 volumes of EtOH to give LFP-II and supernatants were further precipitated by the addition of one volume of EtOH giving LFP-III and with four volumes of EtOH to produce LFP-IV. The precipitates were collected by centrifugation at 4 °C (5,000×g) for 10 min, and lyophilized. The four fractions were refluxed three times with petroleum ether to remove lipids. After filtering, the residues were air-dried and then refluxed again with 80% EtOH and then dissolved in the water. 

### 3.4. Monosaccharide compositions and properties

Carbohydrate contents were determined by the phenol-sulfuric acid method using D-glucose as standard [40]. The protein contents in the polysaccharide fractions were measured according to Bradford’s method, using bovine serum albumin (BSA) as the standard [41]. Gas chromatography (GC) was used for identification and quantication of monosaccharides in the litchi polysaccharide fractions. The polysaccharide (10 mg) was hydrolyzed with 2M trifluoroacetic acid (TFA, 10 mL) at 120°C for 6 hours. Derivatization was then carried out using a trimethylsilylation reagent according to the method of Guentas *et al.* [42]. The trimethylsilylated derivatives were loaded into a HP5 capillary GC column on a HP490D gas chromatograph (America, HP company) equipped with a flame-ionization detector (FID), using inositol as the internal standard. The analysis was performed using the following conditions: H_2_: 16 mL/min; air: 150 mL/min; N_2_: 20 mL/min; injection temperature: 230 °C; detector temperature: 230 °C; column temperature programmed from 130 to 180 °C at 5 °C/min, holding for 2 min at 180 °C, then increasing to 220 °C at 5 °C /min and finally holding for 3 min at 220 °C.

### 3.5. Infrared spectral analysis of the polysaccharides

The IR spectrum of the polysaccharides was determined using a Fourier transform infrared spectrophotometer (Bruker, Germany). The purified polysaccharide was ground with KBr powder and then pressed into pellets for FTIR measurement in the 4,000–500 cm^-1^ frequency range [43].

### 3.6. DPPH scavenging activity

The free-radical scavenging activity was assessed by the method of Yang *et al.* [16] with minor modifications. Briefly, various concentrations of LFP (1 mL) were mixed with 0.2mM DPPH in 95% EtOH (2 mL). After incubation for 30 min at 25 °C in dark, the decrease in the absorbance at 517 nm was measured. Control contained water instead of the LFP solution while blanks contained EtOH instead of DPPH solution. TBHQ was used as a positive control in parallel. The inhibition of DPPH radicals by the LFP samples was calculated according to the following equation: DPPH-scavenging activity (%) = [1-(A_sample517nm_-A_blank517nm_)/ A_control517nm_] × 100.

### 3.7. Superoxide anion-scavenging activity 

The superoxide anion-scavenging activity was measured following the methods of Qi *et al.* [44]. Briefly, various concentrations of LFP (1 mL) were mixed with 0.1M phosphate buffer (pH 7.4, 1 mL) containing NBT (150 μM), PMS (60 μM) and NADH (468 μM). The reaction mixture was incubated at room temperature for 5 min and the absorbance was read at 560 nm. The capability of scavenging superoxide radical was calculated using the following equation: scavenging activity (%) = (1- A_sample560nm_/A_control560nm_) × 100.

### 3.8. Hydroxyl radical scavenging activity 

The hydroxyl radical scavenging activity was measured by consulting the method of Wang *et al.* [45] with minor modifications. The reaction mixture (total volume 4.0 mL) containing the sample solution of various concentrations, EDTA-Fe^2+^ (220 μM), safranine O (0.23 μM), and H_2_O_2_ (60 μM) in potassium phosphate buffer (150 mM, pH 7.4), was incubated for 30 min at 37 °C and the absorbance was read at 520 nm against a blank. Control contained phosphate buffer instead of the H_2_O_2_ while blanks contained distilled water instead of LFP solution. The capability of scavenging hydroxyl radical was calculated using the following equation: Scavenging activity (%) = [(A_sample520nm_-A_blank520nm_)/(A_control520nm_-A_blank520nm_)] × 100.

### 3.9. Ferrous ion chelating ability

Based on the method of Dorma *et al*. [46], the ferrous ion chelating ability of the LFP fractions was determined. Various concentrations of sample solution (0.8 mL) were was mixed with FeCl_2_ (2 mM, 0.4 mL), and then incubated for 5 min at room temperature, and ferroizine (5 mM, 1.6 mL) was added and the mixture shaken well and incubated for 10 min at room temperature. The absorbance was measured at 562 nm against a blank. The ability of all samples to chelated ferrous ion was calculated using the following equation: Chelating ability (%) = (A_control562_ – A_sample562_)/A_control562_ × 100.

### 3.10. Reducing power 

The reducing power was determined according to the method of Duan *et al*. [1] with some modifications. Briefly, various concentrations of sample solution (1 mL) were mixed with 0.2 mM sodium phosphate buffer (pH 6.6, 2.5 mL) and 1% potassium ferricyanide (2.5 mL). The mixture was then incubated at 50 °C for 20 min. After 10% trichloroacetic acid (w/v) was added (2.5 mL), the mixture was centrifuged at 3,000 rpm for 10 min. An aliquot (2.5 mL) of the upper layer was mixed with distilled water (2.5 mL) and 0.1% ferric chloride (0.5 mL), and the absorbance at 700 nm was measured. Reducing power was expressed as a percentage of the activity shown by a 1 mM solution of Vitamin C.

## 4. Conclusions 

Our study led to the preparation of four polysaccharide fractions (LPFs) with different carbohydrate contents from litchi pulp. These LFPs exhibited some antioxidant activity. The antioxidant activity of different LFP fractions was determined, including scavenging activity against superoxide and hydroxyl radicals, and cheating ability. Among the different fractions, LFP-III showed the strongest scavenging activity against DPPH radicals, superoxide and hydroxyl radicals and chelating ability. This evaluation may shed the light on a better understanding on the potential of litchi fruit polysaccharides as a functional antioxidant for their high antioxidant activity. LFP-III was chosen for further investigation of its antioxidant activities and mechanisms *in vivo* in our future work. 

## Figures and Tables

**Table 1:** Components of monosaccharide and properties of polysaccharides from pulp of litchi.

	LFP-I	LFP-II	LFP-III	LFP-IV
Protein (wt%)	2.81	1.31	4.24	1.23
Carbohydrate (wt%)	79.21	85.15	93.09	96.77
Sugar components (mol%)				
D-arabinose	1.95	1.00	1.30	1.60
L-rhamnose	2.00	1.20	1.91	1.00
D-ribose	1.00	nd	1.54	nd
D-galactose	2.04	nd	2.13	1.07
D-glucose	1.57	1.47	1.00	1.21

^#^ nd: not detected.
